# The Influence of Moisturizer Co-Application Protocols on In Vitro Penetration of Betamethasone in Porcine Skin

**DOI:** 10.3390/pharmaceutics17070874

**Published:** 2025-07-03

**Authors:** Daiane L. Rost, Geisa N. Barbalho, Jayanaraian F. M. Andrade, Marcilio Cunha-Filho, Guilherme M. Gelfuso, Tais Gratieri

**Affiliations:** Laboratory of Food, Drugs, and Cosmetics (LTMAC), University of Brasilia (UnB), Brasília 70910-900, DF, Brazil; daianeluizarost@yahoo.com.br (D.L.R.); geisabarbalho@gmail.com (G.N.B.); mjayanaraian@gmail.com (J.F.M.A.); marciliofarm@hotmail.com (M.C.-F.); gmgelfuso@unb.br (G.M.G.)

**Keywords:** atopic dermatitis, betamethasone dipropionate, cutaneous drug delivery, moisturizers, topical corticosteroids

## Abstract

**Background/Objectives:** The treatment of atopic dermatitis frequently involves using a topical corticosteroid and a moisturizer. While the sequential application of these products is a common dermatological practice, their influence on drug penetration remains poorly understood. There is no clear evidence on how hydration, application sequence, and massage affect cutaneous drug delivery. Hence, this study aimed to evaluate the effects of formulation type, moisturizer composition, application sequence, and mechanical stimulation on betamethasone dipropionate (BET) cutaneous penetration. **Methods:** Two commercial formulations (cream and ointment) of BET were evaluated in different experimental conditions, including drug application combined with moisturizers (Cetaphil^®^, as an emollient; Nivea^®^, as an occlusive) pre- or post-application, with or without a 30 s massage. In vitro skin penetration assays were conducted for 12 h using porcine skin mounted in modified Franz diffusion cells. BET levels were extracted from the skin layers and quantified by HPLC. **Results:** The cutaneous BET penetration was strongly influenced by the application sequence, type of moisturizer, and mechanical stimuli. Pre-application of an occlusive or emollient moisturizer, followed by 30 s physical stimuli, significantly enhanced drug retention in the stratum corneum. For the cream, pre-application of moisturizers followed by massage notably increased BET levels in both the stratum corneum and viable skin. Conversely, post-application of moisturizers hindered BET absorption. The ointment showed limited penetration across all conditions, with no drug detected in the viable skin. **Conclusions:** The results showed pre-hydrating the skin, combined with a 30 s massage, was the best strategy for BET diffusion into the skin following cream administration. The formulation type and the order of application directly influence the effectiveness of drug therapy and the topical absorption of BET.

## 1. Introduction

Atopic dermatitis results from a complex triad of factors: skin barrier dysfunction, immune system dysregulation, and changes in the skin microbiome [1,2]. It is characterized by chronic or recurrent inflammation, leading to lesions such as erythema, papules, vesicles, scales, and crusts, often accompanied by intense pruritus [3,4].

One of the main structural alterations observed in atopic dermatitis is the depletion of ceramides in the stratum corneum (SC) [5,6]. This deficiency compromises the skin barrier function, affecting the penetration of active compounds, which must traverse the SC to reach the viable epidermis. Cutaneous penetration into the dermis without systemic absorption is ideal for achieving a therapeutic effect and avoiding systemic exposure [7].

Atopic dermatitis is the most prevalent chronic dermatological skin condition, affecting mainly children, approximately 13%, but also 5% of adults worldwide [8]. A recent study showed that atopic dermatitis prevalence increased by 20.02% from 1990 to 2021 [9]. Although it is not a life-threatening condition, it has a major impact on the quality of life of patients.

To effectively treat atopic dermatitis, topical corticosteroids such as betamethasone must penetrate beyond the SC and reach the viable epidermis and superficial dermis; i.e., viable skin (VS). This is crucial because the primary inflammatory processes responsible for the disease’s symptoms, such as redness, itching, and swelling, occur in these deeper tissue layers. Inflammatory cells and mediators are located within the epidermis and superficial dermis, and the corticosteroid needs to interact directly with these cellular components to exert its anti-inflammatory effects [10].

But, although the skin barrier in atopic dermatitis is often compromised, leading to increased permeability, it still acts as a significant barrier to drug penetration, especially if the skin is not yet fissured. In fact, before fissuring, atopic skin often becomes thickened and leathery (lichenified) [11,12], further hindering the effective penetration of topical agents. Therefore, the SC represents a significant barrier, hindering the passive diffusion of hydrophilic and lipophilic drugs [13,14]. The water content of the SC modulates carrier permeability by influencing diffusion through the intercellular lipid matrix [15]. This is particularly relevant for corticosteroids, which are widely used to manage atopic dermatitis. Ensuring effective drug delivery of these agents is critical, as it may improve treatment efficacy and reduce the need for prolonged corticosteroid use, which is a key therapeutic goal aimed at minimizing adverse effects such as skin thinning and cutaneous atrophy [16,17].

Current treatment often involves corticosteroids in combination with moisturizers, which are recommended to restore skin barrier function and enhance drug penetration [5,18]. Lipids, especially ceramides, are essential for maintaining skin hydration by retaining water in the intercellular spaces and supporting the skin integrity and the skin barrier function [19,20]. Hydration of the SC is known to increase its permeability, thereby facilitating the absorption of drugs [2,21]. But, although moisturizers are widely used to prepare the skin for topical drug application, current guidelines lack consensus on the optimal sequence of application and the impact of mechanical stimulation on the skin [5,16,22]. A study suggested that corticosteroids should be applied at different times than moisturizers, but there is no strong evidence to support this practice in terms of disease progression [7]. Additionally, the effect of massaging the skin to enhance drug absorption is often assumed but has not been sufficiently validated [23,24,25,26].

Hence, this study aimed to investigate the impact of pre-moisturizing the skin with two commercially available moisturizers—Cetaphil^®^, an emollient formulation, and Nivea^®^, an occlusive formulation—on the cutaneous penetration of betamethasone dipropionate (BET). We also assessed the effect of different application sequences relative to the corticosteroid, incorporating mechanical stimulation, i.e., 30 s massage, as a variable. Special attention was given to the differences between cream and ointment formulations. By investigating these factors, this study aimed to provide valuable insights into optimizing atopic dermatitis’ topical treatment.

## 2. Material and Methods

### 2.1. Material

BET cream (0.5 mg/g, EMS, São Paulo, Brazil) and ointment (0.64 mg/g, Candicort^®^, Aché, Brazil) were selected as model corticosteroid formulations. Commercial moisturizers included Cetaphil^®^ Moisturizing Cream (Galderma Laboratories, Dallas, TX, USA) and Nivea^®^ Milk Lotion for Dry to Very Dry Skin (Beiersdorf AG, Hamburg, Germany). Phosphoric acid was purchased from Sigma Aldrich (Steinheim, Germany). Methanol and acetonitrile of HPLC grade were acquired from J.T. Baker (Phillipsburg, NJ, USA). Monobasic sodium phosphate and dibasic sodium phosphate were sourced from Vetec (Rio de Janeiro, Brazil) and used to prepare a phosphate-buffered saline (PBS, pH 7.4). Scotch No. 845 Book Tape was purchased from 3 M (St. Paul, MN, USA). Water was purified using a Milli-Q system (Millipore, MA, USA) with a 0.22 µm pore end filter. Pre-cleaned hydrophobic filters with a diameter of 22 mm and a pore size of 0.45 µm were obtained from Analítica (São Paulo, Brazil).

#### 2.1.1. Excipients of BET Formulations

(a) BET dipropionate cream: Non-ionic self-emulsifying wax, liquid petrolatum, decyl oleate, propylene glycol, phenoxyethanol, ethyl paraben, methylparaben, butylparaben, propylparaben, simethicone, and water.
(b) BET dipropionate ointment: Petrolatum and polyethylene.

#### 2.1.2. Composition of Moisturizers

(a) Emollient Moisturizer (Cetaphil^®^ Moisturizing Cream): Cetaphil Moisturizing Cream is an oil-in-water (O/W) emulsion composed of aqua, glycerin, petrolatum, dicaprylyl ether, dimethicone, glyceryl stearate, cetyl alcohol, *Helianthus annuus* seed oil, PEG-30 stearate, panthenol, niacinamide, *Prunus amygdalus* dulcis oil, tocopherol, tocopheryl acetate, pantolactone, dimethiconol, acrylates/C10–30 alkyl acrylate crosspolymer, carbomer, propylene glycol, BHT, disodium EDTA, benzyl alcohol, phenoxyethanol, sodium hydroxide, citric acid. FIL.1765.V00.
(b) Occlusive Moisturizer (Nivea^®^ Cream): This Nivea moisturizer is a water-in-oil (W/O) emulsion composed of aqua, paraffinum liquidum, glycerin, isododecane, isopropyl palmitate, PEG-40 sorbitan perisostearate, cera microcristallina, polyglyceryl-3 diisostearate, *Prunus amygdalus* dulcis oil, sodium hyaluronate, tocopherol, magnesium sulfate, sodium citrate, citric acid, tocopheryl acetate, potassium sorbate, ethylhexylglycerin, linalool, limonene, geraniol, benzyl alcohol, citronellol, alpha-isomethyl ionone, benzyl benzoate, BHT, parfum.

#### 2.1.3. Porcine Skin

The porcine ear skin was obtained from a local abattoir immediately after slaughter (Suíno Bom Alimentos Ltd., Brasilia, Brazil) from animals intended for human consumption. The skin-obtaining process was conducted according to main ethical guidelines and regulatory standards [27,28,29]. Briefly, the ears were obtained immediately after the animal was slaughtered and before the scalding process. The entire skin was removed from the external region of the ear with the help of a scalpel, and, thus, it was separated from its fatty layer with the help of scissors. The clean skin excised from porcine ears was stored in a vacuum package at −20 °C for a maximum of 1 month before use.

### 2.2. In Vitro Skin Penetration Test

Full-thickness (approximately 2.0 mm) porcine ear skin was used in all experiments. In vitro penetration tests were conducted with a skin disc mounted in a conforming modified Franz-type diffusion cell (diffusion area = 1.3 cm^2^) for 12 h. Prior to the beginning of the tests, the skin was left at room temperature for approximately 30 min for equilibration. The receptor chamber was filled with 15 mL PBS (pH 7.4) and continuously stirred at 300 rpm. The temperature was maintained at 32 ± 1 °C using a water bath. After 12 h, the residual product was carefully removed with purified water. The SC was removed by 15 successive tape-stripping procedures [30]; the tapes were applied only on top of the diffusion area. Finally, the remaining skin (VS) was cut into pieces with scissors. Both tape strips and the skin pieces were extracted with magnetic agitation in methanol for 24 h. After this period, all samples were filtered using a 0.45 µm hydrophobic membrane filter into a vial for posterior BET quantification. Each experiment was performed in sextuplicate (*n* = 6).

#### Experimental Design

**Figure 1:**
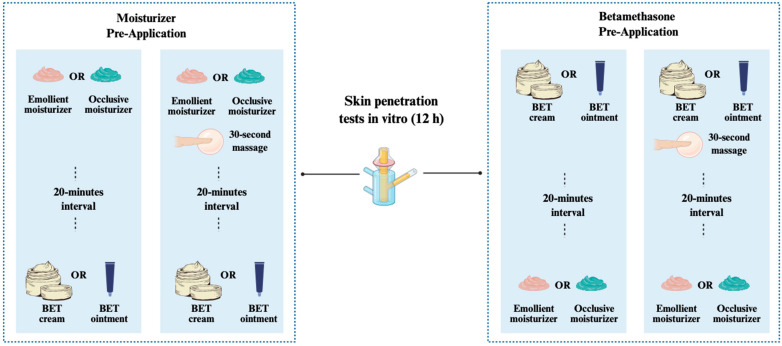
Schematic representation of penetration test conditions involving variation in formulation type, moisturizer (occlusive and emollient moisturizers), and application technique with or without massage, totaling 16 penetration variations. All treatments were conducted before placing the skin samples in the Franz-type diffusion cells.

Figure 1 summarizes the conditions of each skin penetration test. Twenty different conditions were tested, including 4 controls. The controls consisted of BET application (cream or ointment) directly to the skin, followed by a 30 s massage or not for 12 h.

BET cream or ointment was applied in a standardized dose of 600 µL using a syringe without the needle and spread gently over the diffusion area without occlusion. Such an amount was enough to cover the diffusion area of the skin discs with a uniform layer of the formulation. The study was performed by following an infinite dose regimen, in which case the evaporation or diffusion into and through the skin that occurs in the time of the tests is considered negligible [31], allowing for the comparison among different protocols. The moisturizers were also applied in the same amount of 600 µL.

### 2.3. Analytical Method

BET quantification was performed using a reverse-phase High-Performance Liquid Chromatography (HPLC) system (Shimadzu LC-20AT, Kyoto, Japan), with a C18 column (250 mm × 4.6 mm, 5 µm, Supelco^®^, Bellefonte, Pennsylvania, PA, USA) as the stationary phase. The mobile phase consisted of phosphoric acid (1 mM, pH 3.0) and acetonitrile (47:53 *v*/*v*), with a 1.0 mL/min flow rate and a sample injection volume of 10 µL. The detection wavelength was set at 245 nm. The method was previously validated according to ICH Q2(R1) guidelines for linearity (0.2–40 µg/mL, y = 37,551x + 17,350, r = 0.9998), and the detection and quantitative limits were 0.396 µg/mL and 1.2 µg/mL, respectively. Recovery (%) from the SC was 99.0 ± 3.6% and from remaining skin was 93.0 ± 0.6%. The method was also considered precise (RSD < 2%) and specific.

### 2.4. Statistical Analysis

Results were expressed as mean ± standard deviation (SD). Statistical comparisons between groups were conducted using two-way ANOVA followed by Tukey’s post hoc test. Significance was established at 0.05. All analyses were conducted on GraphPad Prism 10 (GraphPad Software, San Diego, CA, USA).

## 3. Results and Discussion

**Figure 2:**
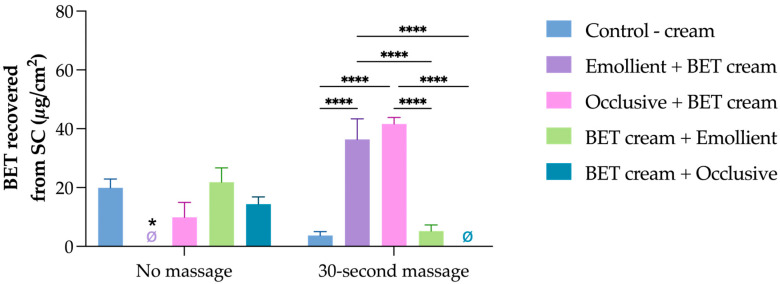
Penetration of betamethasone dipropionate (BET) in the SC following 10 distinct application protocols using a cream-based formulation. Protocols varied by the sequence of BET and moisturizer application (occlusive lotion or emollient-rich cream) and the presence or absence of massage. * *p* = 0.05; **** *p* < 0.0001.

Ten different experimental conditions were evaluated by applying the BET cream formulation (Figure 2). First, controls applied the BET cream directly onto the skin with or without a 30 s massage. The passive control resulted in a BET accumulation in the SC (19.3 ± 3.41 µg/cm^2^) higher (*p* < 0.05) than the formulation that was massaged into the skin (4.16 ± 1.14 µg/cm^2^). This indicates that the mechanical stimuli favored BET diffusion to other skin layers. Indeed, when the cream was massaged, BET reached VS, generating an accumulation of 0.52 ± 0.20 µg/cm^2^, which did not occur in the passive delivery.

When the occlusive moisturizer was pre-applied, followed by BET cream, the moisturizer prevented BET from penetrating the SC, and drug retention was 9.9 ± 5.1 µg/cm^2^, which was lower than the levels achieved with the control. When the pretreatment was performed with the emollient-rich cream, it yielded levels below BET’s quantification limit. It is possible that interactions between the drug, the vehicle, and the lipid components of the cream reduced BET release from the formulation, thereby limiting its penetration into the skin.

Pre-application of the BET formulations did not present statistical differences compared to the control (Figure 2).

The application order relevance was more evident in the mechanically stimulated samples. When the emollient was previously massaged into the skin before BET cream, it resulted in an accumulation of almost 7-fold higher drug levels than when the order was inverted, i.e., when the BET cream was massaged into the skin, followed by the emollient moisturizer application. It was observed that BET also managed to reach the vs. (0.70 ± 0.42 µg/cm^2^) under these conditions.

BET accumulation in the SC was even higher when the occlusive moisturizer was pre-applied. It resulted in an accumulation of 33.3 ± 2.2 µg/cm^2^, while BET was not quantified when the BET formulation was massaged first, followed by the occlusive moisturizer application. In this condition, there was no BET quantification in VS.

These findings challenge conventional assumptions about occlusive formulations and emphasize that penetration outcomes depend not just on formulation type but also on the order in which products are applied. These findings also highlight the importance of incorporating massage techniques, as they can facilitate diffusion beyond the SC.

**Figure 3:**
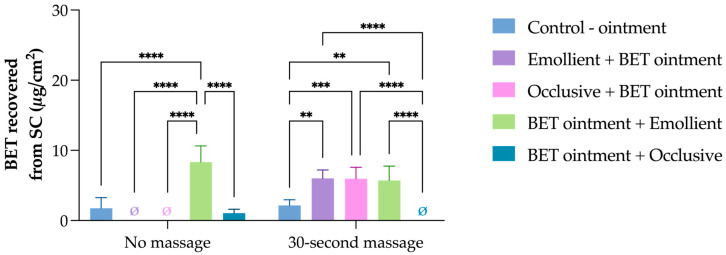
Penetration of betamethasone dipropionate (BET) in the SC following 10 distinct application protocols using an ointment-based formulation. Protocols varied by the sequence of BET and moisturizer application (occlusive lotion or emollient-rich cream) and by the presence or absence of massage. ** *p* = 0.0019, *** *p* = 0.0009, and **** *p* < 0.0001.

To evaluate whether similar procedures would yield comparable outcomes, an ointment formulation was also tested under the same experimental conditions (Figure 3). In this context, pretreatment with either the occlusive lotion or the emollient-rich cream did not enhance drug penetration. For all conditions, BET retention in the SC was consistently lower compared to the BET cream formulation. This behavior is consistent with the ointment’s anhydrous, petrolatum-rich composition, which provides high occlusivity but limits drug release and diffusion across the SC.

For BET ointment, the application of the drug without any moisturizer (control) resulted in a BET accumulation of 1.8 ± 1.5 µg/cm^2^, which was nearly 10-fold lower than that of the BET cream control in the same experimental conditions. When the moisturizers were pre-applied to the skin, no BET was quantified in the SC.

Inverting the order of application, when BET ointment was applied, and an emollient was applied 20 min later, generated the highest BET accumulation (8.33 ± 2.30 µg/cm^2^) among this group, which was statistically superior to all the other approaches. BET ointment followed by the occlusive moisturizer resulted in only 1.06 ± 0.56 µg/cm^2^.

Massage combined with BET ointment application also generated lower retention levels compared to BET cream in the same conditions. When the ointment was directly massaged into the skin, SC retention was limited to 2.2 ± 0.8 µg/cm^2^. In the experiments where the drug ointment was massaged before the occlusive lotion was applied, it hampered deeper drug absorption. In contrast, using emollient moisturizers after BET maintained drug retention in the SC (6.0 ± 1.6 µg/cm^2^).

In the reverse sequence, applying the emollient-rich cream 20 min after the drug application resulted in an SC retention of 6.01 ± 1.20 µg/cm^2^, which was not statistically different (*p* > 0.05) from the use of an occlusive moisturizer in the same conditions (5.96 ± 1.62 µg/cm^2^). Notably, BET was not detected in the vs. in any ointment condition, regardless of application order or massage.

Topical corticosteroid formulations differ significantly in their excipient profiles, directly influencing drug penetration. BET ointment is a water-free formulation composed predominantly of petrolatum, which forms a layer of highly occlusive matrix that limits water evaporation from the SC, thereby increasing skin hydration and enhancing drug residence time on the skin surface [16,32]. Additionally, the ointment contains polyethylene, which serves primarily as a thickening agent but also has emollient properties, which also contribute to its barrier-forming and water-repellent properties, further enhancing occlusion and reducing transepidermal water loss [33,34]. Moreover, their generally lower water content requires fewer preservatives, which is an important advantage when applied to damaged or sensitive skin [35].

In contrast, BET cream is an oil-in-water emulsion containing penetration enhancers such as propylene glycol and decyl oleate, which increase drug solubility and facilitate diffusion through the skin. Usually, it also contains petrolatum, which contributes to occlusivity, but in lower concentrations than those found in ointments [36].

Moisturizers play a critical role not only in cosmetic skincare but also in relieving symptoms associated with chronic skin conditions. Dryness is one of the most uncomfortable symptoms experienced by patients with chronic dermatoses and can exacerbate disease progression [37,38,39].

Consumer acceptance of moisturizers depends on various sensorial attributes, including appearance, initial feel upon application, spreadability, and the level of residual greasiness after use [40]. Creams are the most common cosmetic formulation used for skin hydration [32]. Regarding their mechanism of action, moisturizers are mainly classified as occlusive or emollient types [41].

Occlusive moisturizers form a hydrophobic film over the epidermis, preventing water loss and maintaining hydration. Petrolatum, a hydrocarbon oil, is the most effective occlusive agent, followed by mineral oil and paraffin [36]. These substances are generally oily and also act as emollients, imparting softness and smoothness to the skin [32]. The selected occlusive moisturizer, Nivea Milk, contains a combination of occlusive agents such as paraffinum liquidum (mineral oil), microcrystalline wax, and isopropyl palmitate, which help to limit water evaporation by forming a hydrophobic film. It also includes emollients like sweet almond oil and *Prunus amygdalus* dulcis oil, which improve skin softness and texture.

Emollient moisturizers are formulations designed to soften and smoothen the skin by replenishing surface lipids and filling in the gaps between corneocytes in the SC. These products improve skin texture and flexibility while reinforcing the barrier function, which helps to reduce flaking and irritation. By forming a light, protective film over the skin, emollient moisturizers minimize transepidermal water loss and help maintain hydration, especially in dry or compromised skin [42]. Their use is particularly important in dermatological conditions such as atopic dermatitis, where restoring the skin barrier is a key therapeutic goal. The selected emollient moisturizer, Cetaphil^®^, includes water, glycerin, propylene glycol, panthenol, niacinamide, and other components known for their hygroscopic and barrier-restoring properties.

From the results, it can be hypothesized that BET is diffusing preferably to the moisturizers’ bases rather than the SC. Evidence for this assumption is in the “no massage” group, because when the moisturizers were pre-applied, no BET was quantified in the SC from the BET ointment. As BET is a lipophilic drug (log *P* = 3.6), it is expected to have a high affinity for the oil contents of the ointment, therefore in this case BET remained in the oitment and partition to the skin was low; however, when the BET cream was applied in the same conditions, BET was quantified when the occlusive moisturizer was pre-applied. In such a scenario, BET diffused from the cream base to the occlusive base, but not the emollient one, with a higher water content.

The results found from the “30 s massage” samples also corroborate this hypothesis. When the moisturizers were pre-applied, the physical stimuli enhanced their penetration into the skin, and the BET, especially from the cream base, diffused to the moisturizer that interacted with the SC, resulting in high BET accumulation in the SC. When the application order was inverted, a significant drop occurred in the BET cream group, with low BET quantification when it was combined with the emollient and no quantification at all when the occlusive moisturizer was used (Figure 2). This means that when the moisturizer was “on top”, BET partitioned to the formulation rather than the skin “below”. In the BET ointment group, when BET formulation was pre-applied, it still resulted in some accumulation in the SC in combination with the emollient moisturizer, but not with the occlusive one, probably due to BET diffusion to the moisturizer, resulting in no BET quantification in the SC (Figure 3).

Consequently, in the massaged samples, the order of application was critical, especially in combination with the BET cream. Indeed, this mechanical action can lead to a faster penetration rate and increase the amount of medication retained within the skin tissue [43]. Beyond physical disruption, massage may also locally increase skin temperature and blood flow, both of which are known to promote drug absorption by boosting molecular movement and potentially altering skin structures to allow deeper penetration [24,25,26].

Studies investigating massage often standardize the duration of the mechanical stimuli to ensure consistent drug delivery [44]. While the ideal massage duration can vary based on the drug, its vehicle, and the treated skin area, longer or repeated massages may theoretically enhance penetration by extending contact time and friction, but they can also generate abrasion to the skin [23]. Therefore, a 30 s massage duration was chosen to balance the potential enhancement of penetration with tolerability and ease of application, aiming to minimize the risk of skin damage. In clinical practice, the choice of application protocol is typically guided by the healthcare provider, taking into account the product’s formulation and the patient’s response, aiming to maximize the therapeutic benefit while minimizing adverse effects.

The results highlighted how formulation properties and physical manipulation interact to modulate skin absorption, reinforcing the importance of protocol design in topical therapy. It is important to note, however, that a key limitation of this study is the use of intact skin models. In clinical conditions such as atopic dermatitis, where the skin barrier is compromised, BET penetration into skin layers may be greater due to the altered integrity of the SC. Also, the procedure of applying the BET formulations before placing the skin on the Franz cell could lead to BET penetration in more areas than just the diffusion area of the cell. However, the analyses were based on control samples performed in sextuplicate, which went through the same treatment and, thus, were subjected to the same effect, making the comparisons possible.

Finally, to further corroborate these results, clinical tests are in order. For example, the tape-stripping method was employed in vivo to assess the dermatopharmacokinetics of BET [45]. This kind of study is suitable to investigate the amount of BET penetration into skin in a real-life scenario, as it can estime the rate and extent of drug permeation following topical application. It is also useful to determine the efficacy and safety of a formulation [46], or, in this case, a protocol. This would be one more step towards making atopic dermatitis treatment more effective.

## 4. Conclusions

These findings demonstrate that the cutaneous absorption of BET is significantly influenced by formulation characteristics, vehicle composition, application sequence, and mechanical stimulation. The cream formulation consistently outperformed compared to the ointment, achieving enhanced SC retention and measurable concentrations within viable epidermal layers exclusively when combined with massage application—a penetration profile not observed with the ointment vehicle. Optimal drug retention from the cream was achieved through pre-application skin hydration followed by a 30 s mechanical massage, establishing this protocol as the most effective approach for maximizing topical bioavailability. These findings highlight the critical role of application technique in topical corticosteroid therapy and suggest that pre-hydration and mechanical stimulation may serve as simple, non-invasive strategies to improve therapeutic efficacy in conditions such as atopic dermatitis.

## Data Availability

Data can be provided by the authors upon reasonable request.
